# Tardive dyskinesia with clozapine dose reduction or withdrawal dyskinesia?

**DOI:** 10.4103/0253-7613.75686

**Published:** 2011-02

**Authors:** Samir Kumar Praharaj

**Affiliations:** Department of Psychiatry, Kasturba Medical College, Manipal, Karnataka, India

Sir,

Shrivastava *et al*.[1] have reported an improvement of tardive dyskinesia (TD) with addition of clozapine that exacerbated with its dose reduction (from 200 to 150 mg/day). The patient had developed TD [score 7 on abnormal involuntary movement scale (AIMS)] while being treated with depot fluphenazine injection. It should be noted that the effect of depot injection lasts for a long period and its elimination half-life is longer than that of oral preparations.[2] Thus, it is possible that the index case might have developed withdrawal dyskinesia because of decrease in serum levels of fluphenazine[3], and not clozapine, which has lower affinity for D_2_ receptors. There are even case reports of TD induced or worsened by clozapine therapy.[4–6] Nevertheless, clozapine still remains a viable treatment option for antipsychotic-induced TD,[7] as well as withdrawal dyskinesias,[8] as a maintenance treatment for long periods.[9]

TD was originally caused by fluphenazine. With clozapine (200 mg) treatment for 1 month, symptoms of TD were reduced, and when the dose of clozapine was decreased to 150 mg, symptoms reemerged. Half-life of fluphenazine deconate i.m., which the patient was receiving, is 6–9 days and under multiple dosing, the mean elimination half-life is increased to 14 days.[1] In our case, symptoms of TD reduced after 2 weeks.

Clozapine can improve or worsen TD, and we observed it to improve. The patient is presently maintained on clozapine 200 mg/day without any reemergence of symptoms for last 20 months.
